# Toward an Open Analysis Ecosystem for *Plasmodium* Genomic Epidemiology

**DOI:** 10.4269/ajtmh.25-0418

**Published:** 2026-06-23

**Authors:** Shazia Ruybal-Pesántez, Jorge Amaya-Romero, Sophie Bérubé, Nicholas F. Brazeau, Mouhamadou F. Diop, Nicholas Hathaway, Jason A. Hendry, Kirsty McCann, Kathryn Murie, Maxwell Murphy, Karamoko Niaré, Jody Phelan, Stephen F. Schaffner, Alfred Simkin, Aimee R. Taylor, Bryan Greenhouse, Amy Wesolowski, Robert Verity

**Affiliations:** ^1^ MRC Centre for Global Infectious Disease Analysis, School of Public Health, Imperial College London, London, United Kingdom;; ^2^ Instituto de Microbiología, Universidad San Francisco de Quito, Quito, Ecuador;; ^3^ Department of Immunology and Infectious Disease, Harvard T.H. Chan School of Public Health, Harvard University, Boston, Massachusetts, USA;; ^4^ Department of Biostatistics, University of Florida, Gainesville, Florida, USA;; ^5^ Physician-Scientist Training Program, Duke University Hospital, Durham, North Carolina, USA;; ^6^ The Gambia at London School of Hygiene and Tropical Medicine, Medical Research Council Unit, Fajara, The Gambia;; ^7^ Department of Medicine, University of Massachusetts Chan Medical School, Worcester, Massachusetts, USA;; ^8^ Max Planck Institute for Infection Biology, Berlin, Germany;; ^9^ Centre for Innovation in Infectious Disease and Immunology Research (CIIDIR), The Institute for Mental and Physical Health and Clinical Translation (IMPACT), School of Medicine, Deakin University, Geelong, Australia;; ^10^ EPPIcenter Program, Division of HIV, ID, and Global Medicine, University of California, San Francisco, California, USA;; ^11^ Department of Pathology and Laboratory Medicine, Brown University, Providence, Rhode Island, USA;; ^12^ London School of Hygiene and Tropical Medicine, London, United Kingdom;; ^13^ Infectious Disease and Microbiome Program, Broad Institute of Harvard and MIT, Cambridge, Massachusetts, USA;; ^14^ Institut Pasteur, Université Paris Cité, Paris, France;; ^15^ Department of Epidemiology, Johns Hopkins Bloomberg School of Public Health, Baltimore, Maryland, USA

## Abstract

Major advances in *Plasmodium* sequencing approaches, bioinformatic pipelines, and data analysis tools have provided valuable insights into malaria epidemiology from parasite genomic data. However, translating genetic data into actionable information for decision-makers remains a challenge. Significant barriers limit the integration of these advances into a functional data analysis ecosystem that produces standardized, interpretable results for use by national malaria control programs. The *Plasmodium* Genomic Epidemiology network convened 18 subject matter experts across 15 institutions at the Reproducibility, Accessibility, Documentation, and Interoperability Standards Hackathon in 2023 to identify available analysis tools, evaluate software standards, improve documentation, and outline workflows. Eight use cases for genomic data were identified, and a subset was developed into analysis workflows comprising a series of connected functionalities. Software tools were then mapped against functionalities to outline a modular approach to data analysis for these use cases. In addition to outlining workflows, a set of objective criteria was developed for evaluating software standards. A total of 40 *Plasmodium* genomic analysis tools were identified, 22 of which were prioritized for software standards evaluation. Additional tutorials were developed for 10 tools in the form of reproducible code applied to shared datasets. These resources are available on PGEforge (mrc-ide.github.io/PGEforge), a new community resource that serves as a central, open repository for current and future resources for malaria genomic data analysis.

## THE CENTRAL ROLE OF DATA ANALYSIS IN MALARIA GENOMICS

**Figure 1.**
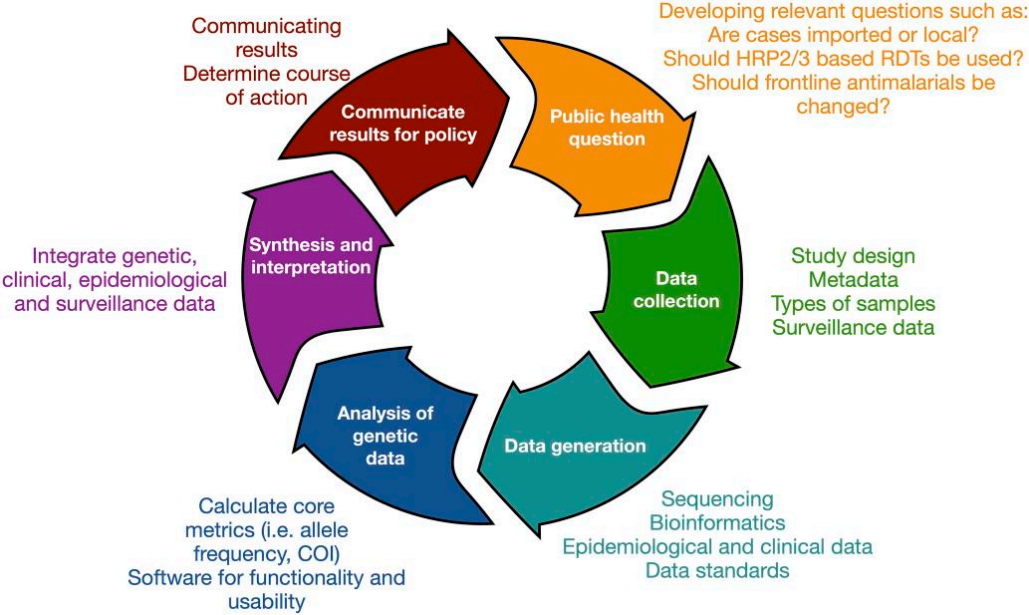
Model for an ideal *Plasmodium* genomic epidemiology framework involving public health stakeholders and scientists. This framework outlines the iterative cycle of malaria genomic epidemiology, integrating public health priority questions, data collection and generation, genetic data analysis, and synthesis and interpretation to inform malaria control and elimination policy. The process begins with defining public health questions relevant to national malaria control and elimination programs, such as whether cases are imported or locally acquired, whether histidine-rich protein 2 (HRP2)-based rapid diagnostic tests should continue to be used, or whether frontline antimalarial drugs remain effective. Next, data collection involves appropriate study design and sample types, as well as contextual data such as demographic information or travel history. Subsequently, data generation will involve sequencing and bioinformatic processing, as well as epidemiological and clinical data, including light microscopy and polymerase chain reaction testing for malaria diagnosis. Robust data standards would enable the harmonization of downstream analysis of genetic data. This stage includes deriving key metrics from genetic data, such as allele frequencies and the complexity of infection, which requires accessible analysis software. The results are then synthesized and interpreted by integrating genetic, clinical, epidemiological, and surveillance data to extract relevant insights and determine appropriate courses of action. Findings are then communicated to public health decision-makers and program officers through a variety of formats, including reports, dashboards, and policy briefs, among others. This will then guide intervention strategies and inform further research questions, thereby continuing the cycle.

In the two decades since the first *Plasmodium falciparum* genome was sequenced,^1^ the malaria research community has developed a wide array of methods for generating insights into malaria epidemiology and transmission dynamics from parasite genetic data. Laboratories can now sequence whole genomes or obtain deep coverage of many genomic targets from small volumes of blood. Advanced bioinformatic pipelines can detect minority alleles present in polyclonal infections while removing errors. An array of computational tools has been developed to distill critical insights from the data, and a variety of visualization methods have been created to communicate results to stakeholders. Collectively, these advancements provide the foundation for establishing a virtuous cycle linking public health personnel with laboratory and data scientists (Figure 1). This collaboration is essential for designing meaningful studies, generating and analyzing sequence data, and translating results into actionable information. An integrated approach will be critical for responding to new threats, including the emergence and spread of drug and diagnostic resistance, increased transmission due to insecticide resistance and invasive mosquito species, and importation due to human migration. However, several significant barriers currently hinder the integration of these efforts into a cohesive data generation and analysis ecosystem.

Substantial variability in sequencing, bioinformatic methods, and nonstandard data formats creates challenges in harmonizing downstream analysis. This variability, however, should not impede progress; rather, adopting a principled approach to analysis should enable users to select from various data generation options while ensuring the comparability of the final results. In addition, although sequencing approaches are increasingly common, other molecular methods, such as electrophoresis-based genotyping of length-polymorphic markers, remain widely used in malaria-endemic settings, further highlighting the need to accommodate diverse data types. Many analysis tools have been created over the past 15 years to address the challenges specific to analyzing *Plasmodium* genetic data, including ploidy, polyclonality, and recombination, which have motivated *Plasmodium*-specific analytical tool development. However, these tools have not been systematically assessed, and vary widely in their adherence to best practices in software development, for example, Findability, Accessibility, Interoperability, and Reuse (often abbreviated as FAIR) standards^2^ or those proposed by the Public Health Alliance for Genomic Epidemiology.^3^ Moreover, there are few, if any, standards for data exchange between them, limiting interoperability. A unified ecosystem that prioritizes interoperability and accessibility with clear data-sharing guidelines and modular workflows, such as that proposed for public health genomic surveillance more broadly,^4^ is essential to maximize the impact of malaria genomic data. Rather than investing resources in self-contained analysis pipelines, a more sustainable and effective solution would be to create a collaborative, transparent, and open platform for curated, interoperable software that translates genetic and contextual data into meaningful results. The advantages of harmonization are clearly evident in other related fields, such as public health genomic surveillance of antimicrobial resistance, where community-led efforts to evaluate and unify software and data standards have led to widespread adoption and a clear path to improve the interoperability of workflows.^5^

Previous community efforts to define applications of genetic epidemiological methods for answering research questions and informing malaria policy have resulted in seven defined use cases.^6^ Implementing workflows for these applications requires going a step further and outlining the types of analyses needed to translate genetic data to desired results. Understanding which tools are available to robustly perform which functions would aid the development of modular analysis workflows.

As a first step in addressing these needs, the *Plasmodium* Genomic Epidemiology (PlasmoGenEpi) network convened 18 subject matter experts from 14 institutions to catalog and test available software tools, evaluate software standards and usability, improve documentation, and evaluate next steps in the context of key use cases for malaria public health and research. This meeting took place at the 2023 Reproducibility, Accessibility, Documentation, and Implementation Software Hackathon (RADISH23), a 4-day event hosted by Johns Hopkins University. This manuscript outlines the main outputs from this event, which center around the PGEforge website, a new community resource for malaria genomic analysis tools.

## OUTLINING USE CASES AND ANALYSIS WORKFLOWS FOR PLASMODIUM GENOMIC EPIDEMIOLOGY

Based on previous publications^6^^,^^7^ and expert discussion to synthesize use cases based on analysis requirements, eight main use cases were defined for *Plasmodium* genetic data informing malaria control and elimination. For each use case, specific functionalities needed for analysis were identified. For a subset of use cases, the authors then outlined how these functionalities could be assembled into analysis workflows to obtain the required results from the initial data.

### Identifying the molecular mechanism or origin of drug and diagnostic resistance.

Initial studies are needed to link parasite genetic variation to phenotypes of drug and diagnostic resistance, for example, by sequencing parasites alongside corresponding data on in vitro responses to antimalarials or on clinical failure of drugs or diagnostic tests. Related studies can be conducted to identify potential polymorphisms of interest via signals of selection and to examine how they are evolving and spreading at various temporal and spatial scales. Case example: Patients are failing therapy with a currently used antimalarial. The basis of resistance is unknown, and there are no identified molecular markers associated with the resistance phenotype. Whole-genome sequencing of parasites with high and low half maximal inhibitory concentration (IC50) values, as measured using ex vivo assays, is performed to identify polymorphisms associated with the resistance phenotype.

### Monitoring the prevalence and frequency of drug or diagnostic resistance markers.

Once genetic polymorphisms have been associated with resistance phenotypes, they can be used as markers of resistance. Marker prevalence (fraction of infections that contain parasites with the resistance marker) and frequency (relative abundance of the resistance marker in the parasite population) can be monitored to track resistance. Various statistical methods may be needed to estimate prevalence or frequency because polyclonal infections complicate otherwise simple analyses. Case example: Phenotypic resistance to artemisinins associated with a specific single-nucleotide polymorphism (SNP) in the *pfk13* gene has been clearly identified in a region of high transmission intensity. Molecular surveillance for this SNP is performed in neighboring areas. Given the high and varied prevalence of polyclonal infections, allele frequency rather than prevalence is calculated to obtain a consistent measure of the proportion of parasites carrying the mutation of interest and to identify areas at risk of artemisinin resistance.

### Measuring human immune selection on the parasite population.

Identifying areas of the *Plasmodium* genome under immune selection can help identify potential targets for vaccines and related immunologic interventions, such as monoclonal antibodies. Related to this, monitoring sequences of such targets can be useful in the context of large-scale implementation to evaluate for selection and monitor for potential escape mutations. Case example: Monoclonal antibodies targeting Plasmodium falciparum circumsporozoite protein are administered to children in a malaria-endemic area. Sequencing of infections identifies mutations enriched in breakthrough infections. This information is used to identify next-generation antibodies that are active against these sequence variants.

### Classifying outcomes in therapeutic efficacy studies as reinfection, recrudescence, or, in the case of *Plasmodium vivax*, relapse.

A therapeutic efficacy study (TES) conducted via prospective evaluation of patients’ responses to treatment for uncomplicated malaria is the gold standard for assessing the efficacy of antimalarial drugs. Genotyping of participants with infections during follow-up is needed to distinguish whether they failed therapy (recrudescence), were infected again (reinfection), or experienced a relapse of a dormant parasite. Case example: A TES performed in a high-transmission area reveals that 50% of participants have late treatment failure (i.e., infections are detected via microscopy during follow-up in half of patients who previously cleared their parasites). High-resolution genotyping via amplicon sequencing, coupled with rigorous statistical analysis, reveals that only 3% of participants experienced recrudescence, providing evidence that the tested antimalarial remains effective.

### Estimating transmission intensity.

Population-level measures derived from parasite genetic data, like other indicators such as parasite prevalence, may be used to estimate malaria transmission intensity for surveillance purposes, including stratification of interventions or evaluation of their effects. Case example: Routine molecular surveillance in an area without accurate measures of incidence and no recent cross-sectional survey indicates an increase in the complexity of infection (COI; the number of genetically distinct clones within an infection) after a change in formulation for indoor residual spraying. Further investigation reveals an increase in vector density and parasite prevalence, prompting a reconsideration of the vector control strategy.

### Estimating the connectivity and movement of parasites between geographically distinct populations.

Identifying whether and to what extent parasites from one geographic area are moving to another can help predict the spread of malaria, as well as the spread of parasites with specific concerning phenotypes such as drug resistance. This, in turn, can inform planning of the scale and timing of interventions. For example, it may be easier to reduce the burden of malaria in an isolated area compared with one that is more connected to areas of higher transmission. This information may be identifiable by comparing genetic measures between populations. Case example: A district within a malaria-endemic country is being considered for subnational elimination. Molecular investigation, coupled with appropriate statistical modeling, reveals a high degree of parasite flow into a neighboring region where the malaria burden is higher. The elimination strategy is broadened to include burden reduction in the neighboring region in parallel with the intensification of local interventions.

### Classifying malaria cases as locally acquired or imported from another population.

In elimination or pre-elimination settings, understanding if cases are imported is relevant to 1) assessing the feasibility of elimination (i.e., elimination is less feasible in areas with a high number of imported infections that may lead to local transmission) and 2) determining if local elimination has been achieved despite reported cases. Case example: An isolated country is evaluating the feasibility of malaria elimination. Travel history is routinely collected, but data are incomplete, and a significant minority of those completing the history do not report travel, raising concerns about continued local transmission. Analysis of molecular data generated for all cases indicates that nearly all cases are likely to be imported or introduced (i.e., the first generation of local transmission). Thus, it is determined that eliminating local transmission may be feasible via the intensification of targeted local interventions.

### Reconstructing granular patterns of transmission.

In areas of very low transmission, characterizing granular details of transmission events, such as chains of transmission between individuals and households, demographic groups at high risk of being infected with or transmitting malaria, hidden reservoirs of residual transmission, contribution of imported cases to local transmission, and the length of sustained transmission chains may help guide and evaluate targeted elimination strategies. This information may be inferred from studies in which infected people are comprehensively sampled and high-resolution genetic data are used to evaluate relationships among infections. Case example: Sustained, low levels of local transmission have been observed in an area hoping to eliminate malaria. Genetic, temporal, and spatial data are integrated using an appropriate statistical model to identify networks of local transmission. The majority of transmission is identified as being driven by certain demographic groups. These demographic groups are targeted with specific interventions to aid the elimination effort.

Each of these eight use cases requires several analysis functionalities to obtain the desired results from *Plasmodium* genetic and contextual data. A total of 14 distinct analysis functionalities that are required or useful for the eight use cases were identified, with many shared across several use cases (Table 1). For example, estimating COI was identified as required or useful for all eight use cases. This makes intuitive sense because analysis of genetic data from polyclonal infections often needs to be treated differently from that from monoclonal infections. Similarly, the estimating phase (assigning alleles to particular clones within a polyclonal sample), population allele frequencies, and within-host relatedness were identified as useful for the majority of use cases. Thus, having a curated set of validated software tools to perform these functions would support a diverse range of analyses. Other functionalities, such as estimating copy number variation or multi-locus genotype frequencies, were only needed for specific use cases.

**Table 1:** Mapping use cases to core functionalities

Use Case	Analysis Functionality													
	Estimate COI	Phase	Estimate allele prevalence	Estimate allele frequency	Estimate multi-locus genotype prevalence	Estimate multi-locus genotype frequency	Identify signs of selection	Estimate between-host relatedness/genetic distance (e.g., IBD, IBS)	Estimate within-host relatedness/genetic distance (e.g., IBD, IBS)	Infer transmission networks	Infer geographic assignment	Calculate simple genetic metrics (e.g., allelic richness, heterozygosity)	Infer structure/clustering	Estimate copy number variation/deletions
Identifying the molecular mechanism/origin of drug and diagnostic resistance	X	X	X	–	X	–	–	X	X	–	–	–	–	–
Monitoring the prevalence/frequency of drug or diagnostic resistance markers	X	X	X	X	X	X	–	–	–	–	–	–	–	X
Measuring human immune selection on the parasite population	X	X	X	X	–	–	–	X	–	–	–	–	–	–
Classifying outcomes in therapeutic efficacy studies as reinfection, recrudescence, or, in the case of *Plasmodium vivax*, relapse	X	X	–	X	–	–	X	–	X	–	–	–	–	–
Estimating transmission intensity	X	X	–	X	X	–	X	–	X	–	–	X	–	–
Estimating the connectivity and movement of parasites between geographically distinct populations	X	X	–	X	–	–	–	–	X	–	X	X	X	–
Classifying malaria cases as locally acquired or imported from another population	X	X	–	X	–	–	–	–	X	X	X	–	X	–
Reconstructing granular patterns of transmission	X	X	–	X	–	–	–	–	X	X	–	–	–	–

COI = complexity of infection; IBD = identity by descent; IBS = identity by state.

**Figure 2.**
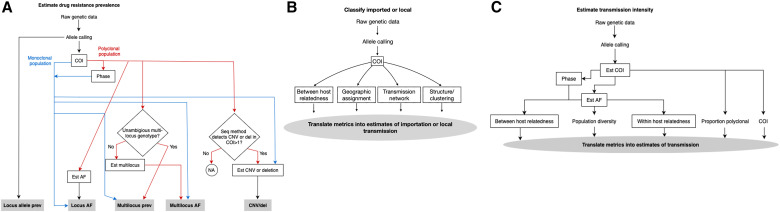
Analysis workflow for key use cases: (**A**) To estimate drug resistance prevalence, raw genetic data are processed through allele calling, and subsequent steps vary depending on the complexity of infection (COI) and whether the population is monoclonal or polyclonal (including monoclonal and polyclonal infections). If the population is monoclonal, allele prevalence (prev) and allele frequencies (AFs) at a single locus can be estimated directly and will be identical. Additionally, multi-locus prev, AFs, and copy number variation (CNV) or deletions can be estimated directly if relevant. If the population is polyclonal and phasing is possible, the analysis follows the same pathway as for a monoclonal population. If phasing is not possible or not performed, additional steps are required, depending on whether unambiguous multi-locus genotypes can be obtained and whether the sequencing method used detects CNV or deletions in polyclonal infections. (**B**) To classify cases as imported or locally acquired, raw genetic data are processed through allele calling, followed by estimation of COI. Then, the estimation of between-host relatedness, geographic assignment, transmission networks, and structure or clustering metrics can be translated into estimates of importation or local transmission. (**C**) To estimate transmission intensity, raw genetic data are processed through allele calling and COI and AF estimation. Subsequent metric estimation involves measuring the proportion of polyclonal infections in the population, between- and within-host relatedness, and population diversity, which can all be collectively translated into estimates of transmission. Alternative analysis pathways may be used depending on whether phasing is possible or performed.

For three example use cases, the authors outlined how analysis workflows might be assembled from the component functionalities (Figure 2). During the process of outlining the workflows, several observations emerged. First, obtaining seemingly straightforward results, such as the frequencies of polymorphisms associated with drug resistance, was more nuanced than generally appreciated. Second, there were often several viable paths to obtaining a given result, based on features of the data (e.g., monoclonal versus polyclonal population) and alternative analytical approaches. Third, within a given path, several software tools might interchangeably provide a given functionality, as discussed in the next section. Alternatively, some workflows are not sequential. They are used to estimate key parameter values (e.g., COI, phase, and frequencies) using data from all samples jointly (i.e., information is borrowed across samples to compensate for relatively uninformative per-sample data) and would fail if polyclonal samples were separated from monoclonal ones. Finally, analysis approaches for some use cases (e.g., estimating transmission intensity) were less mature, and although some analysis steps were clear, others remain poorly defined.

## LANDSCAPING OF AVAILABLE ANALYSIS TOOLS AND MAPPING OF TOOLS TO FUNCTIONALITIES

To begin building a centralized resource for analysis tools to support the eight use cases, the software characteristics of 40 tools were curated and compared (Supplemental Table 1). A total of 22 of these tools were found to pass minimum inclusion criteria for evaluation (see Supplemental Text 1). The characteristics of these tools are shown in Table 2, including implementation type, installation method, platform compatibility, and associated resources (e.g., website, publication, or preprint). It is important to note that this is not an exhaustive list; there are available tools that were not evaluated.

**Table 2:** Tool landscaping matrix

Tool	Characteristic				
	Description (use case and functionalities)	Technical Details	Documentation	Associated Publications	PGEforge Details
MLMOI (Authors: Meraj Hashemi, Kristan Schneider)	- MOI estimation - Allele frequency estimation	- Implementation: R package - Installation: Manual package install from .tar ball (removed from CRAN archive) - Repository/download link: https://cran.r-project.org/src/contrib/Archive/MLMOI/	- Tutorials: available within the package (exact links unavailable) - Package website: unavailable	Hashemi and Schneider 2021 (https://doi.org/10.1371/journal.pone.0261889)	This tool was relegated because of difficulty with installation, as the package has been removed from CRAN. Manual installation instructions were difficult to follow. A tutorial in PGEforge was not developed.
MalHaploFreq (Authors: Ian Hastings)	- Haplotype frequency estimation - Notes: MOI estimation and prevalence estimates are secondary (essentially untested); program does not work on Mac or Linux	- Implementation: .exe file - Installation: Manual download of .exe file - Repository/download link: http://pcwww.liv.ac.uk/hastings/MalHaploFreq/	- Tutorials: unavailable - Package website: unavailable - Archived link: http://pcwww.liv.ac.uk/hastings/MalHaploFreq/	Hastings and Smith 2008 (https://doi.org/10.1186/1475-2875-7-130)	This tool has largely been superseded by MultiLociBiallelicModel, which also estimates prevalence but does not require MOI, can handle more loci, and estimates the MOI from the input data. A tutorial in PGEforge was not developed.
malaria.em (Authors: Xiaohong Li)	- MOI estimation - Haplotype frequency estimation	- Implementation: R package archived on CRAN - Installation: Manual package install from .tar ball (CRAN archive) - Repository/download link: https://cran.r-project.org/src/contrib/Archive/malaria.em/	- Tutorials: available within the package (exact links unavailable) - Package website: unavailable	Li et al. 2007 (https://doi.org/10.2202/1544-6115.1321)	The code for this program was removed from CRAN in 2014, does not appear to be actively maintained, and is no longer available for download other than from archives. A program that does something similar is MultiLociBiallelicModel. As part of PGEforge, the authors have reimplemented this package available at https://github.com/PlasmoGenEpi/malaria.em. A basic tutorial is available on PGEforge.
FreqEstimationModel (Authors: Aimee Taylor)	- MOI estimation; population-level allele frequency estimation; haplotype frequency estimation - Notes: statistical model based on prevalence data; allows for missing data	- Implementation: R package - Installation: Manual download of files or github clone and run files locally - Repository/download link: https://github.com/aimeertaylor/FreqEstimationModel/tree/master	- Tutorials: available within the package (exact links unavailable) - Package website: unavailable	Taylor et al. 2014 (https://doi.org/10.1186/1475-2875-13-102)	A basic tutorial is available on PGEforge.
MultiLociBiallelicModel (Author: Christian Tsoungui Obama)	- MOI estimation - Haplotype frequency estimation	- Implementation: Standalone R scripts - Installation: Git clone and run scripts - Repository/download link: https://github.com/Maths-against-Malaria/MultiLociBiallelicModel	- Tutorials: available within the package (https://github.com/Maths-against-Malaria/MultiLociBiallelicModel/blob/main/src/SNP_MLE.R) - Package website: unavailable	Obama and Schneider 2022 (https://doi.org/10.3389/fepid.2022.943625)	A tutorial is available on PGEforge.
Pfmix (Authors: John O’Brien)	- MOI estimation - Strain mixture proportion inference - Notes: functionalities also include inference of proportion ‘unexplained’ mixture proportions within infections	- Implementation: R package - Installation: Manual package install from .tar ball - Repository/download link: https://github.com/cascobayesian/pfmix/tree/master	- Tutorials: available in the package README (https://github.com/cascobayesian/pfmix/tree/master) - Package website: unavailable	O’brien et al. 2016 (https://doi.org/10.1371/journal.pcbi.1004824)	Because of limited documentation on installation, the authors were unable to install and run the tool. The tool has not been updated in seven years. There is no tutorial available on PGEforge.
THEREALMcCOIL (Authors: Hsiao-Han Chang)	- MOI estimation - Population-level allele frequency estimation - Notes: can be used in categorical (SNP either het or hom) or proportional mode (relative signal intensity for each allele used)	- Implementation: Standalone R scripts (with C++) - Installation: Git clone and compile from source files - Repository/download link: https://github.com/EPPIcenter/THEREALMcCOIL	- Tutorials: unavailable - Package website: unavailable	Chang et al. 2017 (https://doi.org/10.1371/journal.pcbi.1005348)	There is no tutorial available on PGEforge.
McCOILR (Authors: OJ Watson)	- MOI estimation - Population-level allele frequency estimation - Note: this is an R wrapper for THEREALMcCOIL	- Implementation: R package - Installation: devtools - Repository/download link: https://github.com/OJWatson/McCOILR	- Tutorials: available on package website - Package website: https://ojwatson.github.io/McCOILR/articles/introduction.html	Chang et al. 2017 (https://doi.org/10.1371/journal.pcbi.1005348)	This tool is an R wrapper for THEREALMcCOIL; it produces inflated COI estimates as compared with original C++ file from EPPICenter. Presumed seed bug. There is no tutorial available on PGEforge.
Coaif (Authors: Aris Paschalidis and OJ Watson)	- MOI estimation - Note: can get discrete or continuous COI values	- Implementation: R package - Installation: devtools - Repository/download link: https://github.com/bailey-lab/coiaf	- Tutorials: available on package website - Package website: https://bailey-lab.github.io/coiaf/index.html	Paschalidis and Watson et al. 2023 (https://doi.org/10.1371/journal.pcbi.1010247)	There is a tutorial available on PGEforge.
DEploid (Authors: Joe Zhu)	- MOI estimation - MOI proportion estimation - Haplotype estimation	- Implementation: C++ - Installation: Make - Repository/download link: https://github.com/DEploid-dev/DEploid	- Tutorials: available on tool website - Package website: https://deploid.readthedocs.io/en/latest/#	Zhu et al. 2018 (https://doi.org/10.1093/bioinformatics/btx530)	This tool has been superseded by DeploidIBD, which is the same program just run without the IBD flag. There is no tutorial available on PGEforge.
Deploid-r (Authors: Joe Zhu, Jacob Almagro-Garcia, Gil McVean)	- MOI estimation - MOI proportion estimation - Haplotype estimation - Notes: this is an Rcpp wrapper for DEploid	- Implementation: R package - Installation: devtools - Repository/download link: https://github.com/DEploid-dev/DEploid-r	- Tutorials: available on package website - Package website: https://deploid.readthedocs.io/en/latest/index.html	Zhu et al. 2018 (https://doi.org/10.1093/bioinformatics/btx530)	This tool is considered obsolete, as it links to an old version of Deploid. There is no tutorial available on PGEforge.
DEploidIBD (Authors: Joe Zhu)	- MOI estimation - MOI proportion estimation - Haplotype estimation - Within-sample IBD - Notes this is DEploid with “-ibd” flag	- Implementation: C++ - Installation: Make - Repository/download link: https://github.com/DEploid-dev/DEploid	- Tutorials: available on package website - Package website: https://deploid.readthedocs.io/en/latest/index.html	Zhu et al. 2019 (https://doi.org/10.7554/eLife.40845)	There is a tutorial available on PGEforge.
isoRelate (Authors: Lyndal Henden)	Inference of between-sample pairwise IBD in haploid species	- Implementation: R package - Installation: devtools - Repository/download link: https://github.com/bahlolab/isoRelate	- Tutorials: available in package README (https://github.com/bahlolab/isoRelate/blob/master/README.md) - Package website: unavailable	Henden et al. 2018 (https://doi.org/10.1371/journal.pgen.1007279)	There is a tutorial in development for PGEforge.
hmmIBD (Authors: Steve Schaffner)	- Inference of IBD segments between pairs of genomes - Note: includes scripts to extract and thin data	- Implementation: C+ Python scripts - Installation: C compiler - Repository/download link: https://github.com/glipsnort/hmmIBD	- Tutorials: available within the package (link unavailable) - Package website: unavailable	Schaffner et al. 2018 (https://doi.org/10.1186/s12936-018-2349-7)	There is a tutorial available on PGEforge.
dcifer (Authors: Inna Gerlovina)	- COI estimation - Population allele frequency estimation - Estimation of between-sample relatedness (IBD) between polyclonal infections	- Implementation: R package - Installation: CRAN - Repository/download link: https://CRAN.R-project.org/package=dcifer and https://github.com/EPPIcenter/dcifer	- Tutorials: available as vignettes in package and on website - Package website: https://eppicenter.github.io/dcifer/	Gerlovina et al. 2022 (https://doi.org/10.1093/genetics/iyac126)	There is a tutorial available on PGEforge.
Moire (Authors: Max Murphy and Bryan Greenhouse)	- COI estimation - Population-level allele frequency estimation - Note: uses MCMC-based approaches	- Implementation: R package - Installation: devtools - Repository/download link: https://github.com/EPPIcenter/moire	- Tutorials: available as vignettes within the package - Package website: https://eppicenter.github.io/moire/index.html	Murphy and Greenhouse et al. 2024 (https://doi.org/10.1093/bioinformatics/btae619)	There is a tutorial available on PGEforge.
MALECOT (Authors: Bob Verity)	- Inference of population structure - COI estimation - Allele frequency estimation	- Implementation: R package (C++) - Installation: devtools - Repository/download link: https://github.com/bobverity/MALECOT	- Tutorials: available on package website - Package website: https://bobverity.github.io/MALECOT/index.html	N/A	There is no tutorial available on PGEforge; this tool is still under development.
DRpower (Authors: Bob Verity and Shazia Ruybal-Pesántez)	- Estimates power and sample size calculation for *pfhrp2/3* studies - Note: additional interactive web app available	- Implementation: R package (C++) - Installation: devtools - Repository/download link: https://github.com/mrc-ide/DRpower	- Tutorials: available on package website - Package website: https://mrc-ide.github.io/DRpower/	N/A	There is a tutorial available on PGEforge.
estMOI (Authors: Samuel Assefa)	- Estimates multiplicity of infection - Notes: seems that it may be used to be a website and is now a perl script	- Implementation: Perl script - Installation: Manual download - Repository/download link: https://github.com/sammy-assefa/estMOI/tree/master	- Tutorials: available as PDF in github repository (https://github.com/sammy-assefa/estMOI/blob/master/README.pdf) - Package website: https://sourceforge.net/projects/estmoi/	Assefa et al. 2014 (https://doi.org/10.1093/bioinformatics/btu005)	There is no tutorial in PGEforge.
moimix (Authors: Stuart Lee)	- MOI estimation - Note: using heterzyosity, call major allele and thus poly/monoclonal	- Implementation: R package - Installation: BiocManager - Repository/download link: https://github.com/bahlolab/moimix	- Tutorials: available as vignettes within package (https://github.com/bahlolab/moimix/blob/master/vignettes/introduction.Rmd) - Package website: unavailable	N/A	This tool was considered superseded by other MOI estimation tools as it does not estimate MOI; rather, it only differentiates between polyclonal and monoclonal infections. There is no tutorial available in PGEforge.
hmmibdR (Authors: OJ Watson and Steve Schaffner)	This tool is an R wrapper for hmmIBD. To install locally, one needs a C compiler and to run Python scripts to produce input files from raw VCFs.	- Implementation: R package - Installation: This tool is an R wrapper for hmmIBD. To install locally, one needs a C compiler and to run Python scripts to produce input files from raw VCFs. - Repository/download link: https://github.com/OJWatson/hrp2malaRia	- Tutorials: unavailable - Package website: unavailable	Schaffner et al. 2018 (https://doi.org/10.1186/s12936-018-2349-7)	There is no tutorial available in PGEforge.
BRO (Authors: Daniel Larremore)	- Between-sample relatedness estimation with uncertainty - Note: developed for var repertoires; also packaged as an online tool	- Implementation: Python scripts - Installation: git clone and run locally - Repository/download link: https://github.com/dblarremore/BayesianRepertoireOverlap	- Tutorials: available in github repository. - Package website: https://bro.colorado.edu	Larremore 2019 (https://doi.org/10.1371/journal.pcbi.1006898)	This tool is not a packaged open-source tool, so the authors were unable to install it. There is no tutorial available on PGEforge.

COI = complexity of infection; IBD = identity by design; MCMC = markov chain monte carlo; MOI = multiplicity of infection; SNP = single-nucleotide polymorphism; VCF = variant call format.

Among these tools, the majority are implemented as R packages (R Foundation, Vienna, Austria; 14 tools) and others as standalone scripts implemented in R (two tools), C/C++ (International Organization for Standardization, Geneva, Switzerland) or Python (Python Software Foundation, Beaverton, OR, USA; three tools), Perl (The Perl Foundation, Holland, MI, USA; one tool), and a Windows executable file (Microsoft Corp., Redmond, WA, USA; one tool). Most tools are platform-independent and capable of running on multiple operating systems, but one tool is specifically designed for Windows. The installation methods for these tools vary widely: seven require manual installation from source files, six can be cloned and run locally from repositories, six are available via package managers like CRAN (R Foundation) or BiocManager (Bioconductor Project, Buffalo, NY, USA), and three require compilation. For some tools that require compilers, efforts have been made to provide “R wrappers” to decrease installation barriers (e.g., McCOILR [Imperial College London, London, United Kingdom], hmmibdr [Imperial College London]).

The availability of the 14 analysis functionalities was identified (defined in Table 1) in each tool (Table 3). The authors focused on tools that are actively being used because some of the tools were deemed obsolete or superseded, for example, if installation was not possible during the study evaluation, or if they have been superseded by newer tools that perform similar functionalities (these reasons are described in Table 2). The most common functionalities are estimating COI (available in eight different tools), estimating SNP allele frequencies (supported by five tools), and estimating between- and within-host genetic relatedness (available in three and two tools, respectively). Given the number of tools that perform similar functionalities, benchmarking them against standardized datasets will be an important next step to enable validation toward a curated list of best practices. There are also several gaps in current tool capabilities. For example, no existing tools are specifically designed to infer malaria transmission networks, and there is limited support for functionalities such as phasing and geographic assignment, which are covered by only one tool each.

**Table 3:** Tools to analysis functionalities

Tool	Analysis Functionality													
	Estimate COI	Phase	Estimate allele prevalence	Estimate allele frequency	Estimate multi-locus genotype prevalence	Estimate multi-locus genotype frequency	Identify signs of selection	Estimate between-host relatedness/genetic distance (e.g., IBD, IBS)	Estimate within-host relatedness/genetic distance (e.g., IBD, IBS)	Infer transmission networks	Infer geographic assignment	Calculate simple genetic metrics (e.g., allelic richness, heterozygosity)	Infer structure/clustering	Estimate copy number variation/deletions
FreqEstimationModel	X	–	X	X	X	X	–	–	–	–	–	–	–	–
MultiLociBiallelicModel	X	–	X	–	X	–	–	–	–	–	–	–	–	–
THEREALMcCOIL	X	–	–	X	–	–	–	–	–	–	–	–	–	–
Coiaf	X	–	–	–	–	–	–	–	–	–	–	–	–	–
DEploidIBD	X	X	–	–	–	–	–	–	X	–	–	–	–	–
isoRelate	–	–	–	–	–	–	–	X	–	–	–	–	–	–
hmmIBD	–	–	–	X	–	–	–	X	–	–	–	–	–	–
Dcifer	–	–	–	–	–	–	–	X	–	–	–	–	–	–
Moire	X	–	–	X	–	–	–	–	X	–	–	–	–	–
MALECOT	X	–	–	X	–	X	–	–	–	–	X	–	X	–
DRpower	–	–	X	–	–	–	–	–	–	–	–	–	–	X
estMOI	X	–	–	–	–	–	–	–	–	–	–	–	–	–

COI = complexity of infection; IBD = identity by design; IBS = identity by state.

## DEFINING AND EVALUATING USER-ORIENTED AND DEVELOPER-ORIENTED SOFTWARE STANDARDS

Software standards are a critical component in the development and maintenance of reliable, efficient, and interoperable software systems. These standards encompass various aspects of software engineering, including coding practices, documentation, testing, maintenance, and accessibility. Software standards were categorized into two broad groups: 1) those aimed at end-users, and 2) those aimed at developers (Table 4).

**Table 4:** Software standards

Criteria	Type	Notes
User-facing		
Installation instructions exist	binary	–
Usage instructions exist	binary	–
Tutorials exist	binary	Must have an explanation of inputs, outputs, and the test dataset in a worked example
Test data and results available	binary	–
Developer-facing		
Open-source	binary	–
Has software tests	binary	e.g., unit tests
More than 90% code coverage reported	binary	e.g., how much of the source code is tested by automated tests
Clear channels for software maintenance and issues	binary	e.g., GitHub issues, author contact information

**Figure 3.**
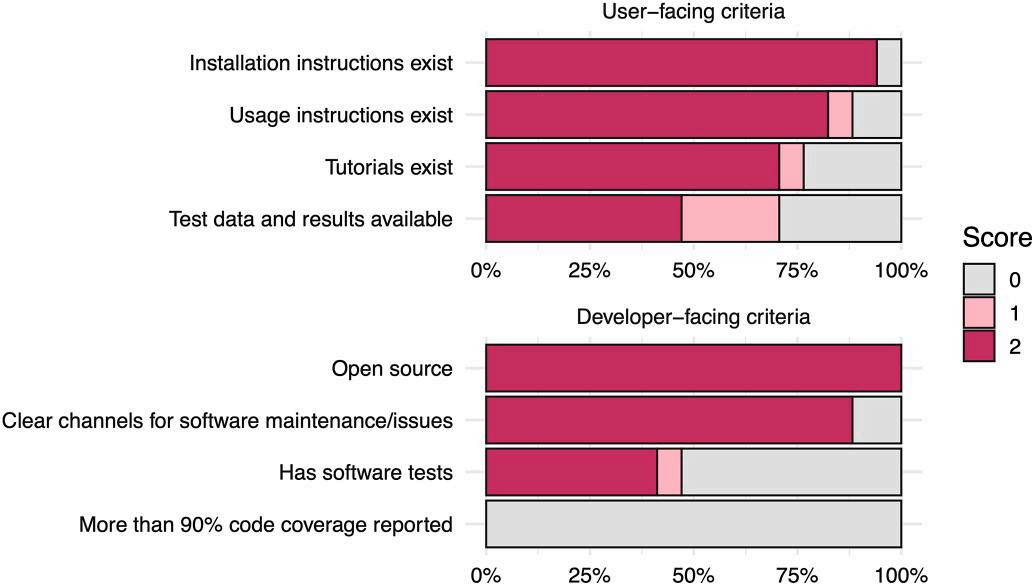
Evaluation of tools based on software standards for user- and developer-facing criteria.

All eight software standards were applied to the 22 tools prioritized from the landscaping exercise (as of August 2024). For each software standard, a scale from 0 to 2 was used, where 0 indicates that the standard was not met, 1 indicates the standard was partially met (for example, if usage instructions exist but are not comprehensive), and 2 indicates the standard was fully met. Overall, user-oriented software standards were generally met more consistently than developer-oriented standards (Figure 3).

Most tools provide basic installation instructions and usage guidance, whereas tutorials and test data sets with expected results were not always available. For developer-oriented software standards, although all tools were open-source and offered reasonably clear channels for software maintenance and issue resolution, few provided adequate testing, and none achieved high code coverage. This indicates a reasonable effort to make tools accessible and open-source for end-users; however, there is still room for improvement in providing comprehensive usage resources, testing, and documentation.

## STREAMLINING INSTALLATION AND MANAGING SYSTEM DEPENDENCIES

Historically, a significant barrier to the adoption of software tools has been the challenges associated with their installation and the management of system dependencies. Although tools may function correctly at the time of publication, differences in operating systems, software versions, and configurations often lead to incompatibilities that hinder successful installation or execution. Hidden system dependencies, in particular, can cause unexpected failures that are difficult to diagnose without advanced knowledge of the underlying software architecture. These obstacles can discourage end users, especially those with limited technical expertise, thereby reducing the accessibility and impact of the tools.

To address this, the authors developed the PlasmoGenEpi r-universe (plasmogenepi.r-universe.dev), a dynamic platform hosting R package repositories. This platform features an automated build process that incorporates platform-specific system dependencies, providing precompiled R packages to end-users and eliminating the need for local compilation. Although this approach significantly improves usability and accessibility, further work on containerization would better facilitate fully reproducible analysis workflows that can operate seamlessly across diverse computational environments.

## DEVELOPMENT OF OPEN-SOURCE COMMUNITY RESOURCES

Community resources are essential for advancing malaria genomics by fostering collaboration, standardization, and accessibility in tool development and data analysis. To address this need, the authors developed PGEforge (mrc-ide.github.io/PGEforge) as a community-driven platform for end-users and developers of malaria genomic tools. PGEforge serves as a centralized hub for identifying and evaluating analysis tools and outlining analysis workflows tailored to key research questions. PGEforge also hosts freely available example genomic datasets in widely used formats, including both whole-genome sequencing and amplicon data, as well as simulated datasets with known ground truth and curated empirical datasets. Additionally, it offers comprehensive, fully reproducible tutorials, initially developed for 10 analysis tools identified during RADISH23, that leverage the publicly available datasets hosted on the platform. This resource is designed to streamline and enhance malaria genomics research through accessibility, standardization, and reproducibility. Importantly, the dynamic nature of this online hub will enable the addition of new developments as they emerge, for instance, additional canonical simulated or empirical datasets, analysis tools and methods, updated software evaluations, use cases, or research questions requiring different analysis workflows, tool benchmarking, or any other relevant community developments. These can be readily proposed by the community and updated directly on the platform.

## NEXT STEPS TOWARD AN OPEN ANALYSIS ECOSYSTEM FOR PLASMODIUM GENOMIC EPIDEMIOLOGY

The efforts described here represent an important step toward improving the usability of analysis tools for malaria genomic epidemiology; however, more work is needed to ensure that useful results can be reliably obtained by a broad range of users. This work includes filling gaps in analysis functionality, formally evaluating the accuracy of tools for parameter estimation, and creating a broader environment that facilitates flexible assembly of tools into accessible workflows. Mapping workflows provides valuable insight into gaps currently underserved by existing software tools. Notable gaps include tools for in silico phasing of genomic data from polyclonal infections, which were identified as potentially useful across all eight defined use cases. Additionally, specialized tools are needed for specific use cases, such as the classification of outcomes in antimalarial TESs.

Where appropriate tools do exist, data on their accuracy are often limited (at best) to an initial evaluation by the original developers. Rigorous benchmarking of tools across different types of data, including assessment of performance in realistic situations that do not satisfy all model assumptions, will allow users and workflow developers to make informed choices on which tools are most appropriate for given applications. Standardized, curated datasets with known truth (e.g., from simulations or controlled experiments, or those freely provided in PGEforge), along with well-defined performance measures, will be an important step in facilitating routine benchmarking. For example, Guo and colleagues conducted a comprehensive benchmarking of the accuracy of tools for estimating identity by descent (IBD) using simulated ground truth data to show the impact of marker density on accurate IBD detection.^8^ Further development of simulation frameworks in which specific parameters can be controlled (e.g., polyclonality, within- and between-host relatedness) and benchmarking work is needed, including updated curation as the available set of tools expands and evolves. This work is currently underway by the PlasmoGenEpi network members.

Aspects important for end-to-end workflows but not addressed during the RADISH23 hackathon include evaluating upstream steps in bioinformatic allele calling and downstream steps in the communication and visualization of results. Choosing an appropriate bioinformatic pipeline to obtain allele calls is important because it determines the type (e.g., SNPs, indels, or microhaplotypes) and quality of data used for subsequent analyses. Several pipelines exist for various types of sequencing data (e.g., built around GATK [Broad Institute, Cambridge, MA, USA] for obtaining SNPs and indels from whole-genome sequencing data^9^ or SeekDeep^10^ [Bailey lab at Brown University] or DADA2^11^ [North Carolina State University, Raleigh, NC, USA] for obtaining microhaplotypes from targeted sequencing data). However, there have been few efforts to perform systematic benchmarking of these pipelines. As with analysis tools, curated ground truth datasets will be useful for improving (e.g., optimizing tuning parameters), evaluating, and comparing pipelines. At the other end of the workflow, thoughtful consideration of how best to summarize and display results would substantially improve their accessibility and, therefore, their public health impact. Providing several options for users, including summary tables and figures (e.g., https://genremekong.org/tools/grcmalaria-r-package-user-guide), potentially utilizing dynamic interfaces (e.g., those described by van Wyck et al.^12^ and Battle et al.^13^), may be a worthwhile investment of resources. Linking all of these components together—allele-calling pipelines, analysis tools, and display of results—is somewhat challenging at present. Creating a set of interoperability standards (e.g., agreed-upon formats for inputs and outputs at various steps) would greatly facilitate assembly of these steps into complete workflows that start with raw genetic and contextual data and return results in interpretable formats. Similarly, creating example workflows (e.g., well-commented scripts with associated documentation and tutorials that connect several disparate tools) can expand access to data analysis for specific use cases.

It will require some upfront effort to optimize, benchmark, and document the many steps required to guide raw sequence data through to meaningful results. However, such an investment is apt to yield large dividends, enabling the harmonization of disparate components created via uncoordinated efforts. The end result would be a collaborative, transparent, and open platform of interoperable software, following the successful lead of this approach for other systems (e.g., QIIME [Northern Arizona University, Flagstaff, AZ, USA], https://qiime2.org/). This model fosters community contributions, promotes healthy competition, and ensures that tools continue to evolve while remaining relevant and applicable. Important considerations for democratizing access to such a platform will include documentation in several relevant languages beyond English and flexibility to install workflows locally or run them remotely on cloud-based platforms (e.g., TERRA, Broad Institute^14^). Future development of the ecosystem should also be focused on how best to visualize complex genetic data outputs, including landscaping and evaluation of existing visualization tools, and on exploring the most appropriate formats for communicating with stakeholders (e.g., policy briefs, dashboards, short reports). In addition, the potential role of artificial intelligence in enhancing and streamlining some of these workflows, for instance, to develop interactive analytical workflow guides, could also be considered in future developments.

In conclusion, a substantial body of work has been produced on analysis tools for *Plasmodium* genomic epidemiology. Thoughtful, targeted improvements to these existing tools in the context of an open ecosystem designed for organized growth have the potential to greatly extend their utility. The authors hope that the resources described here are a useful step in continued efforts toward this goal.

## Supplemental Materials

10.4269/ajtmh.25-0418 Supplemental Materials
