# Supplementary material for: Toward an Open Analysis Ecosystem for Plasmodium Genomic Epidemiology
Source: Am J Trop Med Hyg. 2026 Jun 23;115(2):216–28. doi: 10.4269/ajtmh.25-0418 (PMC13447779; doi:10.4269/ajtmh.25-0418)
Supplement: Supplementary file 1 [file tpmd250418.SD1.pdf]

Supplementary Materials for:

Towards an open analysis ecosystem for *Plasmodium* genomic epidemiology

Shazia Ruybal-Pesántez[1,2], Jorge Amaya-Romero[3], Sophie Bérubé[4], Nicholas F. Brazeau[5], Mouhamadou Fadel Diop[6], Nicholas Hathaway[7], Jason Hendry[8], Kirsty McCann[9], Kathryn Murie[10], Maxwell Murphy[10], Karamoko Niaré[11], Jody Phelan[12], Stephen F. Schaffner[3,13], Alfred Simkin[11], Aimee R. Taylor[14], Bryan Greenhouse[10], Amy Wesolowski[15], Robert Verity[1*]

[1] MRC Centre for Global Infectious Disease Analysis, School of Public Health, Imperial College London, UK
[2] Instituto de Microbiología, Universidad San Francisco de Quito, Ecuador
[3] Department of Immunology and Infectious Disease, Harvard T.H. Chan School of Public Health, Harvard University, USA
[4] Department of Biostatistics, University of Florida, USA
[5] Physician-Scientist Training Program, Duke University Hospital, Durham USA
[6] Medical Research Council Unit The Gambia at London School of Hygiene and Tropical Medicine, The Gambia
[7] Department of Medicine, University of Massachusetts Chan Medical School, USA
[8] Max Planck Institute for Infection Biology, Germany
[9] Centre for Innovation in Infectious Disease and Immunology Research (CIIDIR), The Institute for Mental and Physical Health and Clinical Translation (IMPACT), School of Medicine, Deakin University, Australia
[10] EPPIcenter program, Division of HIV, ID, and Global Medicine, University of California San Francisco, USA
[11] Department of Pathology and Laboratory Medicine, Brown University, USA
[12] London School of Hygiene and Tropical Medicine, UK
[13] Infectious Disease and Microbiome Program, Broad Institute of Harvard and MIT, USA
[14] Institut Pasteur, Université Paris Cité, France
[15] Department of Epidemiology, Johns Hopkins Bloomberg School of Public Health, USA

Contents:

Text S1: Methods

Table S1: Tool landscaping matrix

Table S2: “Nice to have” software standards

Text S1: Methods

Landscaping of available analysis tools

We conducted a landscaping exercise of available tools that are commonly applied to *Plasmodium* genetic data. This included identifying, cataloging, and assessing tools. In order to limit the scope of this exercise during RADISH23, we applied the following constraints:

- Focus on downstream analysis tools. This includes tools whose primary goal is to extract signal from pre-processed data, but does not include tools that are primarily used within upstream bioinformatic steps, such as variant callers and quality filters.
- Focus on *Plasmodium* genetics, including both *P. falciparum* and *P. vivax*. We did not consider applications to mosquito genetics, despite some tools and techniques being applicable to both organisms.
- Focus on tools that are specifically engineered for *Plasmodium*. While there are many generic methods that can be applied to any type of genetic data (e.g. F-statistics and other population genetic metrics), we did not seek to evaluate these tools.

These constraints were necessary in order to make a meaningful contribution within a narrow timeframe, although we strongly advocate for future events expanding the scope of this exercise.

Defining software standards for tool evaluation

When establishing objective criteria for evaluating software standards, we divided our approach into two categories: user-oriented standards and developer-oriented standards.
User-oriented software standards describe a set of best practices and guidelines that prioritize the needs and expectations of end-users. These standards cover interface design, usability, accessibility, and performance, which together create a cohesive and intuitive experience. Although central to commercial software development, user-oriented standards tend to be a blind-spot in academic software development where the incentive structure favors innovation over usability. We identified four standards as critical in promoting a positive user experience (Table 4), along with three characteristics that are desirable but not critical (Supplementary Table 2).

Developer-oriented software standards focus on ensuring that the software development process is efficient, maintainable, and scalable. This is important for the long-term viability of tools, the transparency of methods, and the potential to extend and modify existing frameworks as data and use cases change. We identified four standards as critical in promoting a robust development process (Table 4), along with four characteristics that are desirable but not critical (Supplementary Table 2).

Compiling datasets of common genetic data input formats

We compiled empirical and simulated datasets of commonly used input formats that could be used as a reference set by the community. In addition, these same datasets were used across all tutorials developed during RADISH23, thereby ensuring results were reproducible and comparable between tools. We identified the following key features to ensure appropriateness and utility for downstream analysis tools:

- A subset of monoclonal and polyclonal samples, lab strains, and lab mixtures

- Various formats: VCF files, BAM files, tabular haplotypes in .csv
- A subset of polyclonal samples with within-host relatedness greater than 0
- Some amount of missing data

The data input formats derived were whole genome sequences, microhaplotypes, SNP barcodes, and *pfhrp2/3* gene deletion counts. All are freely available at the PGEforge GitHub repository at https://github.com/mrc-ide/PGEforge/tree/main/data.

Whole genome sequencing data

We derived three VCF files from the MalariaGEN Pf3K project (https://www.malariagen.net/parasite/pf3k), selecting samples from Democratic Republic of Congo (DRC) (n=113), Vietnam (n=97), and laboratory strain mixtures (n=25). Each VCF contains 247,496 high-quality (VQSLOD>6) biallelic SNPs across all fourteen somatic chromosomes. The VCFs are sorted and accompanied by an index file. We provide associated contextual data for the samples, which includes Fws statistics from the MalariaGEN Pf7K dataset (https://www.malariagen.net/sites/default/files/Pf7_fws.txt) (except for the lab mixtures). These are deposited at https://github.com/mrc-ide/PGEforge/tree/main/data/wgs/pf3k.

We also simulated a dataset of samples with varying COI and within-host relatedness. Briefly, a simulated sample with a given COI, *K,* was created by randomly sampling *K* clonal haplotypes (Fws >0.95, [1]) from the Pf3K project (https://www.malariagen.net/parasite/pf3k), assigning these haplotypes to $j \leq K$ infectious bites, and if $j < K$, simulating meiosis with a constant recombination rate of 13.5kbp/cM [2]. For each haplotype, proportions were randomly sampled from a symmetric Dirichlet distribution, and for each locus, read count data was simulated from a Beta-binomial distribution given the allelic proportions. Constant sequencing error was simulated in the allele counts at rates (REF to ALT, 10^{-5}; ALT to REF, 5×10^{-4}) broadly consistent with observation for the included sites. No variant calling error was simulated. In this way, we produced a VCF file with 40 simulated DRC samples, ranging from COI of one to four, with approximately half of the samples having within-host relatedness. Accompanying contextual data is provided as .csv and .bedfiles, which are deposited at https://github.com/mrc-ide/PGEforge/tree/main/data/wgs/simulated/pf3k-DRCongo-simA.

To derive BAM files, we subsetted the VCF calls to only *csp* (PF3D7_0304600), *celTOS* (PF3D7_1133400) and *ama1* (PF3D7_1216600) genes, and provide accompanying contextual data. These are deposited at https://github.com/mrc-ide/PGEforge/tree/main/data/wgs/labisolate_subset.

Microhaplotype data

We provided microhaplotype data from 82 samples from Mozambique and lab strain mixtures from the study by [3]. The microhaplotype panel included 100 targets (91 diversity and 9 drug targets). The lab strain mixtures are from various combinations of seven *P. falciparum* lab strains, with some sequenced in replicate at 4 different parasite densities (10, 100, 1000, 10000 parasites per µl). The datasets and their accompanying contextual data can be found at https://github.com/mrc-ide/PGEforge/tree/main/data/amplicon.

We also simulated a Mozambique dataset to create 100 samples with 50 diverse targets sub-selected from the MAD^4HatTeR panel [4]. Both PCR and Illumina error was simulated using in-house C++ scripts. Starting parasite densities were sampled from previous Mozambique data [3], and PCR was simulated by taking the randomly sampled parasite density and simulating 23 rounds of PCR with a per-base error rate of 3.5e-06 for taq PCR rate and simulating chimeras. Illumina error was introduced by creating a Nextseq 3D7 reference data error profile. Haplotypes were randomly sampled from a previous Mozambique dataset, which was also used to generate a distribution of COI [5].

SNP barcodes

We derived SNP barcode data by subsetting the Pf3k WGS data from the samples from Vietnam and DRC described above to match the 101-SNP barcode panel from [6]. The lab strain and lab mixtures dataset are subsetted to the 101-SNP barcode, with the mixtures created from various combinations of *P. falciparum* lab strains. We provide the accompanying contextual data with information on the genotypes that make up the mixtures and the COI.

We also simulated 101-SNP barcode data for 100 samples (50 from Bangladesh and 50 from Ghana) to include samples with a range of COI, as follows. A simulated sample was created by simulating superinfections by sampling the SNP barcode from each of these countries and selecting COIs based on the observed COIs in each country. Simulations were then created similarly to the details above for the Mozambique dataset but data was sampled from Pf7 dataset [7]. We also provide an additional dataset where we removed indels. The datasets are provided at https://github.com/mrc-ide/PGEforge/tree/main/data/snp_barcode.

pfhrp2/3 gene deletion counts

We provide *pfhrp2/3* gene deletion count data in tabular .csv format from the study by [8] conducted in twelve sites in Ethiopia. The dataset can be found at https://github.com/mrc-ide/PGEforge/tree/main/data/pfhrp2-3_counts.

Tutorial development

For each tool, we completed summary documents, installation aids and tutorials. Ten tools were completed during RADISH23, and guidelines were made available for future tutorial development by anyone in the community. These tutorials provide background information on each analysis tool, installation instructions and a fully worked-through analysis tutorial with code showing how an end user could use the tool to analyze data.

Table S1: All tools landscaping matrix

Note: for easier viewing, also see user-friendly version online here

Tool	Characteristic				
	**Description (use case and unctionalities)**	**Technical details**	**Documentation**	**Associated publications**	**PGEforge details**
**MLMOI**<br><br>*Authors: Meraj Hashemi, Kristan Schneider*	- MOI estimation<br>- Allele frequency estimation	**- Implementation**: R package<br>**- Installation:** Manual package install from .tar ball (removed from CRAN archive).<br>**- Repository/download link**: https://cran.r-project.org/src/contrib/Archive/MLMOI/	- **Tutorials:** available within the package (exact links unavailable)<br>- **Package website:** unavailable	Hashemi and Schneider 2021 (https://doi.org/10.1371/journal.pone.0261889)	This tool was relegated due to difficulty with installation as the package has been removed from CRAN. Manual installation instructions were difficult to follow. A tutorial in PGEforge was not developed.
**MalHaploFreq**<br><br>*Authors: Ian Hastings*	- Haplotype frequency estimation<br>- *Notes: MOI estimation and prevalence estimates are secondary (essentially untested), program does not work on Mac or Linux*	**- Implementation:** .exe file<br>**- Installation:** Manual download of .exe file<br>**- Repository/download link:** http://pcwww.liv.ac.uk/hastings/MalHaploFreq/	- **Tutorials:** unavailable<br>- **Package website:** unavailable<br>- **Archived link:** http://pcwww.liv.ac.uk/hastings/MalHaploFreq/	Hastings and Smith 2008 (https://doi.org/10.1186/1475-2875-7-130)	This tool has largely been superseeded by MultiLociBiallelicModel, which also estimates prevalence but does not require MOI, can do more loci and estimates the MOI from the input data. A tutorial in PGEforge was not developed.
**malaria.em**<br><br>*Authors: Xiaohong Li*	- MOI estimation<br>- Haplotype frequency estimation	**- Implementation**: R package archived on CRAN<br>**- Installation:** Manual package install from .tar ball (CRAN archive)<br>**- Repository/download link**: https://cran.r-project.org/src/contrib/Archive/malaria.em/	- **Tutorials:** available within the package (exact links unavailable)<br>- **Package website:** unavailable	Li et al 2007 (https://doi.org/10.2202/1544-6115.1321)	The code for this program was removed from CRAN in 2014, does not appear to be actively maintained and is no longer available for download other than from archives. A program that does something similar is MultiLociBiallelicModel. As part of PGEforge we have reimplemented this package available at https://github.com/PlasmoGenEpi/malaria.em. A basic tutorial is available on PGEforge.
**FreqEstimationModel**<br><br>*Authors: Aimee Taylor*	- MOI estimation<br>- Population-level allele frequency estimation<br>- Haplotype frequency estimation<br>- *Notes: Statistical model based on prevalence data. Allows for missing data*	**- Implementation**: R package<br>**- Installation:** Manual download of files or github clone and run files locally<br>**- Repository/download link**: https://github.com/aimeertaylor/FreqEstimationModel/tree/master	- **Tutorials:** available within the package (exact links unavailable)<br>- **Package website:** unavailable	Taylor et al 2014 (https://doi.org/10.1186/1475-2875-13-102)	A basic tutorial is available on PGEforge.

**MultiLociBiallelicModel**<br><br>*Authors: Christian Tsoungui Obama*	- MOI estimation<br>- Haplotype frequency estimation	- **Implementation**: Standalone R scripts<br>- **Installation:** Git clone and run scripts<br>- **Repository/download link**: https://github.com/Maths-against-Malaria/MultiLociBiallelicModel	- **Tutorials:** available within the package (https://github.com/Maths-against-Malaria/MultiLociBiallelicModel/blob/main/src/SNP_MLE.R)<br>- **Package website:** unavailable	Obama and Schneider 2022 (https://doi.org/10.3389/fepid.2022.943625)	A tutorial is available on PGEforge.
**pfmix**<br><br>*Authors: John O'Brien*	- MOI estimation<br>- Strain mixture proportion inference<br>- *Notes: functionalities also include inference of proportion 'unexplained' mixture proportions within infections*	- **Implementation**: R package<br>- **Installation:** Manual package install from .tar ball<br>- **Repository/download link**: https://github.com/cascobayesian/pfmix/tree/master	- **Tutorials:** available in the package README (https://github.com/cascobayesian/pfmix/tree/master)<br>- **Package website:** unavailable	O'brien et al 2016 (https://doi.org/10.1371/journal.pcbi.1004824)	Due to limited documentation on installation, we were unable to install and run the tool. The tool has not been updated in seven years. There is no tutorial available on PGEforge.
**THEREALMcCOIL**<br><br>*Authors: Hsiao-Han Chang*	- MOI estimation<br>- Population-level allele frequency estimation<br>- *Notes: can be used in categorical (SNP either het or hom) or proportional mode (relative signal intensity for each allele used)*	- **Implementation**: Standalone R scripts (with C++)<br>- **Installation:** Git clone and compile from source files<br>- **Repository/download link**: https://github.com/EPPIcenter/THEREALMcCOIL	- **Tutorials:** unavailable<br>- **Package website:** unavailable	Chang et al 2017 (https://doi.org/10.1371/journal.pcbi.1005348)	There is no tutorial available on PGEforge.
**McCOILR**<br><br>*Authors: OJ Watson*	- MOI estimation<br>- Population-level allele frequency estimation<br>- *Note: this is an R wrapper for THEREALMcCOIL*	- **Implementation**: R package<br>- **Installation:** devtools<br>- **Repository/download link**: https://github.com/OJWatson/McCOILR	- **Tutorials:** available on package website<br>- **Package website:** https://ojwatson.github.io/McCOILR/articles/introduction.html	Chang et al 2017 (https://doi.org/10.1371/journal.pcbi.1005348)	This tool is an R wrapper for THEREALMcCOIL, it produces inflated COI estimates as compared to original C++ file from EPPICenter. Presumed seed bug. There is no tutorial available on PGEforge.
**coaif**<br><br>*Authors: Aris Paschalidis and OJ Watson*	- MOI estimation<br>- *Note: can get discrete or continuous COI values*	- **Implementation**: R package<br>- **Installation:** devtools<br>- **Repository/download link**: https://github.com/bailey-lab/coiaf	- **Tutorials:** available on package website<br>- **Package website:** https://bailey-lab.github.io/coiaf/index.html	Paschalidis and Watson et al 2023 (https://doi.org/10.1371/journal.pcbi.1010247)	There is a tutorial available on PGEforge.
**DEploid**<br><br>*Authors: Joe Zhu*	- MOI estimation<br>- MOI proportion estimation<br>- Haplotype estimation	- **Implementation**: C++<br>- **Installation:** Make<br>- **Repository/download link**: https://github.com/DEploid-dev/DEploid	- **Tutorials:** available on tool website<br>- **Package website:** https://deploid.readthedocs.io/en/latest/#	Zhu et al 2018 (https://doi.org/10.1093/bioinformatics/btx530)	This tool has been superseded by DeploidIBD, which is the same program just run without the IBD flag. There is no tutorial available on PGEforge.
**Deploid-r**<br><br>*Authors: Joe Zhu, Jacob Almagro-Garcia, Gil McVean*	- MOI estimation<br>- MOI proportion estimation<br>- Haplotype estimation<br>- Notes: this is an Rcpp wrapper for DEploid	- **Implementation**: R package<br>- **Installation:** devtools<br>- **Repository/download link**: https://github.com/DEploid-dev/DEploid-r	- **Tutorials:** available on package website<br>- **Package website:** https://deploid.readthedocs.io/en/latest/index.html	Zhu et al 2018 (https://doi.org/10.1093/bioinformatics/btx530)	This tool is considered relegated as it links to an old version of Deploid. There is no tutorial available on PGEforge.

**DEploidIBD**<br><br>*Authors: Joe Zhu*	- MOI estimation<br>- MOI proportion estimation<br>- Haplotype estimation<br>- Within-sample IBD<br>*- Notes this is DEploid with "-ibd" flag*	**- Implementation**: C++<br>**- Installation:** Make<br>**- Repository/download link**: https://github.com/DEploid-dev/DEploid	- **Tutorials:** available on package website<br>- **Package website:** https://deploid.readthedocs.io/en/latest/index.html	Zhu et al 2019 (https://doi.org/10.7554/eLife.40845)	There is a tutorial available on PGEforge.
**isoRelate**<br><br>*Authors: Lyndal Henden*	- Inference of between-sample pairwise IBD in haploid species	**- Implementation**: R package<br>**- Installation:** devtools<br>**- Repository/download link**: https://github.com/bahlolab/isoRelate	- **Tutorials:** available in package README (https://github.com/bahlolab/isoRelate/blob/master/README.md)<br>- **Package website:** unavailable	Henden et al 2018 (https://doi.org/10.1371/journal.pgen.1007279)	There is a tutorial in development for PGEforge.
**hmmIBD**<br><br>*Authors: Steve Schaffner*	- Inference of IBD segments between pairs of genomes<br>*- Notes: Includes scripts to extract and thin data*	**- Implementation**: C+ Python scripts<br>**- Installation:** C compiler<br>**- Repository/download link**: https://github.com/glipsnort/hmmIBD	- **Tutorials:** available within the package (link unavailable)<br>- **Package website:** unavailable	Schaffner et al 2018 (https://doi.org/10.1186/s12936-018-2349-7)	There is a tutorial available on PGEforge.
**paneljudge**<br><br>*Authors: Aimee Taylor and Pierre Jacob*	- Evaluates the performance of a panel of genetic markers designed to test relatedness<br>*- Note: can also estimate relatedness between monoclonals*	**- Implementation**: R package<br>**- Installation:** devtools<br>**- Repository/download link**: https://github.com/aimeertaylor/paneljudge	- **Tutorials:** available as vignettes within the package<br>- **Package website:** unavailable	LaVerriere et al 2022 (https://onlinelibrary.wiley.com/doi/10.1111/1755-0998.13622)	There is a tutorial available on PGEforge.
**dcifer**<br><br>*Authors: Inna Gerlovina*	- COI estimation<br>- Population allele frequency estimation<br>- Estimation of between-sample relatedness (IBD) between polyclonal infections	**- Implementation**: R package<br>**- Installation:** CRAN<br>**- Repository/download link**: https://CRAN.R-project.org/package=dcifer and https://github.com/EPPIcenter/dcifer	- **Tutorials:** available as vignettes in package and on website<br>- **Package website:** https://eppicenter.github.io/dcifer/	Gerlovina et al 2022 (https://doi.org/10.1093/genetics/iyac126)	There is a tutorial available on PGEforge.
**moire**<br><br>*Authors: Max Murphy and Bryan Greenhouse*	- COI estimation<br>- Population-level allele frequency estimation<br>*- Note: uses MCMC based approaches*	**- Implementation**: R package<br>**- Installation:** devtools<br>**- Repository/download link**: https://github.com/EPPIcenter/moire	- **Tutorials:** available as vignettes within the package<br>- **Package website:** https://eppicenter.github.io/moire/index.html	Murphy and Greenhouse et al 2024 (https://doi.org/10.1093/bioinformatics/btae619)	There is a tutorial available on PGEforge.
**MALECOT**<br><br>*Authors: Bob Verity*	- Inference of population strucure<br>- COI estimation<br>- Allele frequency estimation	**- Implementation**: R package (C++)<br>**- Installation:** devtools<br>**- Repository/download link**: https://github.com/bobverity/MALECOT	- **Tutorials:** available on package website<br>- **Package website:** https://bobverity.github.io/MALECOT/index.html	N/A	There is no tutorial available on PGEforge, this tool is still under development.

**SIMPLEGEN**<br><br>*Authors: Bob Verity, Shazia Ruybal-Pesántez, Isobel Routledge, Sohpie Berube, Daniel Larremore*	- Transmission simulator of malaria epidemiology and parasite genotypes	- **Implementation**: R package (C++)<br>- **Installation:** devtools<br>- **Repository/download link**: https://mrc-ide.github.io/SIMPLEGEN/	- **Tutorials:** available on package website<br>- **Package website:** https://mrc-ide.github.io/SIMPLEGEN/index.html	N/A	There is no tutorial available on PGEforge, this tool is still under development.
**MAGENTA**<br><br>*Authors: OJ Watson*	- Transmission simulator of malaria epidemiology and parasite genotypes<br>*- Note: Implements interventions and a basic spatial model*	- **Implementation**: R package (C++)<br>- **Installation:** devtools<br>- **Repository/download link**: https://github.com/OJWatson/magenta	- **Tutorials:** available on package website<br>- **Package website:** https://ojwatson.github.io/magenta/articles/Introduction.html	Watson et al 2020 (https://doi.org/10.1093/molbev/msaa225)	This tool is a simulator and thus not within the original scope of PGEforge genomic data analysis tools. There is no tutorial available on PGEforge.
**DRpower**<br><br>*Authors: Bob Verity and Shazia Ruybal-Pesántez*	- Estimates power and sample size calculation for *pfhrp2/3* studies<br>- Note: additional interactive web app available	- **Implementation**: R package (C++)<br>- **Installation:** devtools<br>- **Repository/download link**: https://github.com/mrc-ide/DRpower	- **Tutorials:** available on package website<br>- **Package website:** https://mrc-ide.github.io/DRpower/	N/A	There is a tutorial available on PGEforge.
**pixelate**<br><br>*Authors: Aimee Taylor, James Watson and Caroline Buckee*	- pixelates geographic maps according to levels of uncertainty	- **Implementation**: R package<br>- **Installation:** CRAN<br>- **Repository/download link**: https://github.com/aimeertaylor/pixelate	- **Tutorials:** available as vignettes on package github repository<br>- **Package website:** unavailable	Taylor et al 2020 (https://doi.org/10.48550/arXiv.2005.11993)	There is no tutorial available on PGEforge.
**estMOI**<br><br>*Authors: Samuel Assefa*	- Estimates multiplicity of infection<br>- *Notes:* seems that it may be used to be a website and is now a perl script	- **Implementation:** Perl script<br>- **Installation:** Manual download<br>- **Repository/download link:** https://github.com/sammy-assefa/estMOI/tree/master	- **Tutorials:** available as PDF in github repository (https://github.com/sammy-assefa/estMOI/blob/master/README.pdf)<br>- **Package website:** https://sourceforge.net/projects/estmoi/	Assefa et al 2014 (https://doi.org/10.1093/bioinformatics/btu005)	There is no tutorial in PGEforge.
**forward-dream**<br><br>*Authors: Jason Hendry and Gil McVean*	- Simulates malaria transmission and evolution	- **Implementation:** Python<br>- **Installation:** conda<br>- **Repository/download link:** https://github.com/JasonAHendry/fwd-dream	- **Tutorials:** unavailable<br>- **Package website:** unavailable	Hendry et al 2021 (https://doi.org/10.1371/journal.pcbi.1009287)	This tool is a simulator and thus not within the original scope of genomic data analysis tools. There is no tutorial available in PGEforge.
**moimix**<br><br>*Authors: Stuart Lee*	- MOI estimation<br>- Note: using heterzyosity, call major allele and thus poly/monoclonal	- **Implementation:** R package<br>- **Installation:** BiocManager<br>- **Repository/download link:** https://github.com/bahlolab/moimix	- **Tutorials:** available as vignettes within package (https://github.com/bahlolab/moimix/blob/master/vignettes/introduction.Rmd)<br>- **Package website:** unavailable	N/A	This tool was considered superseded by other MOI estimation tools as it does not estimate MOI, rather it only differentiates between poly/monoclonal infections. There is no tutorial available in PGEforge.

**hrp2malaria**<br><br>*Authors: OJ Watson*	- Individual based malaria transmission model for hrp2 deletion simulation	- **Implementation:** R package<br>- **Installation:** devtools<br>- **Repository/download link:** https://github.com/OJWatson/hrp2malaRia	- **Tutorials:** available in README<br>- **Package website:** unavailable	Watson et al 2017 (https://doi.org/10.1371/journal.pcbi.1009287)	This tool is a simulator and thus not within the scope of genomic data analysis tools. There is no tutorial available on PGEforge.
**hmmibdR**<br><br>*Authors: OJ Watson and Steve Schaffner*	This tool is an R wrapper for hmmIBD, to install locally need C compiler and to run python scripts to produce input files from raw VCFs.	- **Implementation:** R package<br>- **Installation:** This tool is an R wrapper for hmmIBD, to install locally need C compiler and to run python scripts to produce input files from raw VCFs.<br>- **Repository/download link:** https://github.com/OJWatson/hrp2malaRia	- **Tutorials:** unavailable<br>- **Package website:** unavailable	Schaffner et al 2018 (https://doi.org/10.1186/s12936-018-2349-7)	There is no tutorial available in PGEforge.
**MIPanalyzer**<br><br>*Authors: Bob Verity, OJ Watson and Nick Brazeau*	- Filtering of MIP data, PCA, pairwise genetic distances	- **Implementation**: R package<br>- **Installation:** devtools<br>- **Repository/download link**: https://github.com/mrc-ide/MIPanalyzer	- **Tutorials:** available on package website<br>- **Package website:** https://mrc-ide.github.io/MIPanalyzer/index.html	N/A	There is no tutorial available on PGEforge.
**PlasmoSim**<br><br>*Authors: Bob Verity*	- Simulation of genetic data (SNPs, haplotypes) or ancestry (IBD)<br>*- Notes: Options for human movement between demes.*	- **Implementation**: R package (beta)<br>- **Installation:** devtools<br>- **Repository/download link**: https://github.com/mrc-ide/PlasmoSim	- **Tutorials:** available on package website<br>- **Package website:** https://mrc-ide.github.io/PlasmoSim/	N/A	This tool is a simulator and thus not within the scope of genomic data analysis tools. There is no tutorial available on PGEforge.
**polySimIBD**<br><br>*Authors: Nick Brazeau and Bob Verity*	- Forwards in-time simulation of *Plasmodium* population genetics	- **Implementation**: R package (beta)<br>- **Installation:** devtools/remotes<br>- **Repository/download link**: https://github.com/nickbrazeau/polySimIBD	- **Tutorials:** available as vignettes in the package (https://github.com/nickbrazeau/polySimIBD/tree/master/vignettes)<br>- **Package website:** unavailable	N/A	This tool is a simulator and thus not within the scope of genomic data analysis tools. There is no tutorial available on PGEforge.
**discent**<br><br>*Authors: Nick Brazeau and Bob Verity*	- Estimates deme inbreeding spatial coefficient (DISC)	- **Implementation**: R package (beta)<br>- **Installation:** devtools/remotes<br>- **Repository/download link**: https://github.com/nickbrazeau/discent	- **Tutorials:** available as vignettes in the package<br>- **Package website:** unavailable	N/A	This tool is still under development. There is no tutorial available on PGEforge.
**SNP-slice**<br><br>*Authors: Nianqiao Ju, Jiawei Liu, Qixin He*	- Reconstruction of SNP haplotypes<br>- COI estimation<br>*- Note: Reconstruction of SNP haplotypes allows for inference of haplotype/strain frequencies*	- **Implementation**: Standalone R scripts<br>- **Installation:** git clone and run locally<br>- **Repository/download link**: https://github.com/nianqiaoju/snp-slice	- **Tutorials:** unavailable<br>- **Package website:** unavailable	Ju et al 2024 (https://doi.org/10.1093/bioinformatics/btae344)	As part of PGEforge we have reimplemented this package available at https://github.com/PlasmoGenEpi/snp.slicer. A basic tutorial is available on PGEforge.
**BRO**<br><br>*Authors: Daniel Larremore*	- Between-sample relatedness estimation with uncertainty<br>*- Note: developed for var repertoires. Also packaged as an online tool*	- **Implementation:** Python scripts<br>- **Installation:** git clone and run locally<br>- **Repository/download link:** https://github.com/dblarremore/BayesianRepertoireOverlap	- **Tutorials:** available in github repository.<br>- **Package website:** https://bro.colorado.edu	Larremore 2019 (https://doi.org/10.1371/journal.pcbi.1006898)	This tool was not a packaged open-source tool so we were unable to install. There is no tutorial available on PGEforge.

**Pv3Rs**<br><br>*Authors: Yong See Foo and Aimee Taylor*	- Between-sample relatedness estimation with uncertainty<br>- *Note: compute_posterior() function could also be used for Pf by setting prior probability of relapse=0*	**- Implementation: R package**<br>**- Installation:** CRAN<br>**- Repository/download link:** **https://github.com/aimeertaylor/Pv3Rs**	**- Tutorials:** available on package website<br>**- Package website:** https://aimeertaylor.github.io/Pv3Rs/	Foo et al 2025 (https://doi.org/10.1093/bioinformatics/btaf643)	This tool was still under development during the hackathon. There is no tutorial available on PGEforge.
**Anti-malarial MCMC Bayesian Algorithm**<br><br>*Authors: Mateusz Plucinski*	- Estimate TES outcomes from genotyping data<br>- *Note: This version is NOT designed for NGS data but length polymorphism data using capillary electrophoresis. There is a version with minor modifications adapted from this; Monica Golumbeanu @SwissTPH hopefully getting a clean version on a GitHub repo soon.*	**- Implementation:** Python scripts<br>**- Installation:** git clone and run locally<br>**- Repository/download link:** https://github.com/GTJuniorDesign0100-2020/anti-malarial-MCMC-bayesian-algorithm	**- Tutorials:** available in github repository README<br>**- Package website:** https://github.com/GTJuniorDesign0100-2020/anti-malarial-MCMC-bayesian-algorithm/blob/master/README.md	Plucinski et al 2015 (https://doi.org/10.1128/aac.00072-15)	This tool was still in development during the hackathon. There is no tutorial available on PGEforge.
**AmpSeq**<br><br>*Authors: Jorge Amaya, Ruchit Panchal, Phillip Schwabl, Pablo Manrique*	- Denoising and amplicon assembly	**- Implementation:** Python scripts<br>**- Installation:** git clone and run locally<br>**- Repository/download link:** https://github.com/broadinstitute/malaria-amplicon-pipeline/tree/main	**- Tutorials:** available in github repository<br>**- Package website:** https://github.com/broadinstitute/malaria-amplicon-pipeline/tree/main	LaVerriere et al 2022 (https://onlinelibrary.wiley.com/doi/10.1111/1755-0998.13622)	This tool focuses on upstream bioinformatic data processing and is thus not within the scope of PGEforge genomic data analysis tools. There is no available tutorial in PGEforge.
**AmpSim**<br><br>*Authors: Nick Hathaway*	- Simulating PCR and sequencing noise	**- Implementation:** C++<br>**- Installation:** git clone, docker, vagrant<br>**- Repository/download link:** https://github.com/nickjhathaway/elucidator	**- Tutorials:** unavailable<br>**- Package website:** unavailable	N/A	This tool is a simulator and thus not within the scope of genomic data analysis tools. There is no tutorial available on PGEforge.
**rehh**<br><br>*Authors: Alexander Klassmann, Mathieu Gautier, Renaud Vitalis*	- Estimating and plotting extended haplotype homozygosity (EHH)<br>- Estimation of integrated haplotype score (iHS)	**- Implementation:** R package<br>**- Installation:** devtools<br>**- Repository/download link:** https://cran.r-project.org/web/packages/rehh/vignettes/rehh.html	**- Tutorials:** available on gitlab<br>**- Package website:** https://gitlab.com/oneoverx/rehh	Gautier and Vitalis 2012 (https://doi.org/10.1093/bioinformatics/bts115); Gautier et al 2016 (https://doi.org/10.1111/1755-0998.12634)	This tool focuses on population genetics, not specifically focused on *Plasmodium* and thus was not within the original scope of PGEforge *Plasmodium* genomic data analysis tools during the hackathon.
**Hierfstat**<br><br>*Authors: Jerome Goudet, Thibaut Jombart, Zhian N. Kamvar, Eric Archer, Olivier Hardy*	- Different types of F-statistics (per locus and pairwise)	**- Implementation:** R package<br>**- Installation:** devtools, CRAN<br>**- Repository/download link:** https://github.com/jgx65/hierfstat	**- Tutorials:** available as package vignettes<br>**- Package website:** https://cran.r-project.org/web/packages/hierfstat/vignettes/hierfstat.html	Goudet 2004 (https://doi.org/10.1111/j.1471-8286.2004.00828.x)	This tool focuses on population genetics, not specifically focused on *Plasmodium* and thus was not within the original scope of PGEforge *Plasmodium* genomic data analysis tools during the hackathon.
**MicroHaploHet**<br><br>*Authors: Karamoko Niare*	- Microhaplotype heterozygosity<br>- Microhaplotype-based PCA	**- Implementation:** Bash and Python scripts<br>**- Installation:** git clone<br>**- Repository/download link:** unavailable	**- Tutorials:** unavailable<br>**- Package website:** unavailable	N/A	This tool is still under development. There is no tutorial available on PGEforge.

Table S2: "Nice to have" software standards

Criteria	Type	Notes
**User-facing**		
Informative error handling	binary	
Multiple languages for tutorials	binary	
Uses standard data input formats	binary	Eg decreases the need for the user to wrangle data into required input format
**Developer-facing**		
Minimal dependencies	binary	
Computationally efficient	binary	
Modular code	binary	Eg split into functions
Well-annotated code	binary	

References

1. Manske M, Miotto O, Campino S, Auburn S, Almagro-Garcia J, Maslen G, O'Brien J, Djimde A, Doumbo O, Zongo I, Ouedraogo J-B, Michon P, Mueller I, Siba P, Nzila A, et al., 2012. Analysis of Plasmodium falciparum diversity in natural infections by deep sequencing. *Nature 487*: 375–379
2. Miles A, Iqbal Z, Vauterin P, Pearson R, Campino S, Theron M, Gould K, Mead D, Drury E, O'Brien J, Ruano Rubio V, MacInnis B, Mwangi J, Samarakoon U, Ranford-Cartwright L, et al., 2016. Indels, structural variation, and recombination drive genomic diversity in Plasmodium falciparum. *Genome Res 26*: 1288–1299
3. Tessema SK, Hathaway NJ, Teyssier NB, Murphy M, Chen A, Aydemir O, Duarte EM, Simone W, Colborn J, Saute F, Crawford E, Aide P, Bailey JA, Greenhouse B., 2020. Sensitive, Highly Multiplexed Sequencing of Microhaplotypes From the Plasmodium falciparum Heterozygome. *J Infect Dis 225*: 1227–1237
4. Aranda-Díaz A, Vickers EN, Murie K, Palmer B, Hathaway N, Gerlovina I, Boene S, Garcia-Ulloa M, Cisteró P, Katairo T, Semakuba FD, Nsengimaana B, Gwarinda H, García-Fernández C, Da Silva C, et al., 2024. Sensitive and modular amplicon sequencing of Plasmodium falciparum diversity and resistance for research and public health
5. da Silva C, Boene S, Datta D, Rovira-Vallbona E, Aranda-Díaz A, Cisteró P, Hathaway N, Tessema S, Chidimatembue A, Matambisso G, Nhama A, Macete E, Pujol A, Nhamussua L, Galatas B, et al., 2023. Targeted and whole-genome sequencing reveal a north-south divide in P. falciparum drug resistance markers and genetic structure in Mozambique. *Commun Biol 6*: 619
6. Chang H-H, Wesolowski A, Sinha I, Jacob CG, Mahmud A, Uddin D, Zaman SI, Hossain MA, Abul Faiz M, Ghose A, Sayeed AA, Ridwanur Rahman M, Islam A, Karim MJ, Kamar Rezwan M, et al., 2019. Mapping imported malaria in Bangladesh using parasite genetic and human mobility data
7. MalariaGEN, Abdel Hamid MM, Abdelraheem MH, Acheampong DO, Ahouidi A, Ali M, Almagro-Garcia J, Amambua-Ngwa A, Amaratunga C, Amenga-Etego L, Andagalu B, Anderson T, Andrianaranjaka V, Aniebo I, Aninagyei E, et al., 2023. Pf7: an open dataset of Plasmodium falciparum genome variation in 20,000 worldwide samples. *Wellcome Open Res 8*: 22
8. Feleke SM, Reichert EN, Mohammed H, Brhane BG, Mekete K, Mamo H, Petros B, Solomon H, Abate E, Hennelly C, Denton M, Keeler C, Hathaway NJ, Juliano JJ, Bailey JA, et al., 2021. Plasmodium falciparum is evolving to escape malaria rapid diagnostic tests in Ethiopia. *Nature Microbiology 6*: 1289–1299
